# Introducing multiple-choice questions to promote learning for medical students: effect on exam performance in obstetrics and gynecology

**DOI:** 10.1007/s00404-020-05758-1

**Published:** 2020-08-31

**Authors:** Sebastian M. Jud, Susanne Cupisti, Wolfgang Frobenius, Andrea Winkler, Franziska Schultheis, Sophia Antoniadis, Matthias W. Beckmann, Felix Heindl

**Affiliations:** Department of Gynecology and Obstetrics, Erlangen University Hospital, Friedrich Alexander University of Erlangen–Nuremberg, Universitätsstrasse 21–23, 91054 Erlangen, Germany

**Keywords:** Teaching, Multiple choice, Testing, Gynecology, Obstetrics, Lecture

## Abstract

**Purpose:**

Testing is required in medical education. The large number of exams that students face requires effective learning strategies. Various methods of improving knowledge retention and recall have been discussed, two of the most widely evaluated of which are test-enhanced learning and pause procedures. This study investigated the effect of voluntary multiple-choice questions on students’ performance.

**Methods:**

In a prospective study from April 2013 to March 2015, 721 students were randomly assigned to receive supplementary online material only (control group) or additional multiple-choice questions (investigative group) accompanying lectures. Their performance in the final exam was evaluated.

**Results:**

A total of 675 students were ultimately included, with 299 randomly assigned to the investigative group and 376 to the control group. Students in the investigative group scored significantly better in relation to grades and points (2.11 vs. 2.49; 33 vs 31.31; *p* < 0.05). The effect declined over time.

**Conclusion:**

This is the first study of the use of voluntary multiple-choice questions to improve medical students’ performance. The results support test-enhanced learning and the feasibility of implementing multiple-choice questions in lectures.

## Background and introduction

The Medical Licensing Regulations (*Ärztliche Approbationsordnung,* ÄAppO) for medical students in Germany require testing in several medical specialties (§ 27 ÄAppO), particularly in larger specialties such as general medicine, surgery, internal medicine, and obstetrics and gynecology. Each university is able to regulate independently how, and at what point in time during courses, these examinations are carried out.

Testing is familiar to all teachers and is mainly used for assessment purposes such as measuring students’ learning achievements or assigning grades in educational courses. Testing is also widely accepted in medical education, mainly using multiple-choice questions, as a way of evaluating knowledge at university level—not only in Germany but also all over the world. State medical examinations use testing to determine that those who have passed are permitted to provide medical care [1]. Feedback is only provided by the grade achieved, or even just by a pass/fail result [2].

A new aspect of testing in education is currently attracting more and more attention. There is a strong consensus that testing enhances learning and the long-term retention of information (“test-enhanced learning,” TEL). More specifically, individuals who are tested regularly are able to remember and recall information better than participants who study the same material for an equivalent amount of time without testing [3–5]. However, other trials have demonstrated that continuous testing has an impact only on students’ motivation, but not on their final test results [6]. Independently of the test format, the greatest transfer of learning effect is reported for questions involving application and inference and problems involving medical diagnoses [7]. Other studies have also analyzed the results after testing and found no difference between the groups, although testing was seen to be more effective relative to long-term performance [8].

The classic method for knowledge transference in medical teaching is through lectures. This type of teaching has been an increasing topic of interest in studies evaluating the impact of different teaching methods, in efforts to improve acceptance by students and to enhance students’ performance. A recent study evaluated variables associated with achievement in higher education. Using “conceptually demanding learning tasks,” presenting information in a clear way, and relating it to the students were found to be strongly associated with achievement. Students with high achievement levels also appreciated having clear learning goals and an ability to provide feedback [9]. The technique of including a pause procedure during lectures, to provide an opportunity for discussion among the students, also improves performance in multiple-choice questions [10]. However, this technique is time-consuming [11]. A combination of a pause procedure and in-class answering of multiple-choice questions was also associated with a large improvement in another trial [12].

Students face a large number of examinations at the end of each semester. Strategies for effective learning methods are therefore of particular interest [13].

## Aim

Introducing a pause procedure into lectures in our institution would reduce the amount of information conveyed and is time-consuming. To improve knowledge retention among students and to provide an effective learning strategy without the disadvantages of pause procedures, a study was designed to evaluate the use of voluntary responses to multiple-choice questions at home during the course of a semester, to include the effects of test-enhanced learning, providing feedback, and relating the information to the students.

## Methods

### Study design

Lecture in gynecology and obstetrics were obligatory, lasted one semester and the main topics of gynaecological oncology, general gynecology, reproductive medicine and obstetrics were taught. The course ended with an obligatory exam about the mentioned topics.

All students who enrolled for the lecture-course and written examination in gynecology and obstetrics at Erlangen University Hospital, Friedrich Alexander University of Erlangen–Nuremberg, between April 2013 and March 2015 were invited to take part in this prospective randomized trial. After consent, all participants were randomly assigned to two groups.

The control group received access to online learning materials only, while the investigative group had the opportunity, in addition to being able to access the online learning material, to complete three multiple-choice questions on each lecture (with a total of 17 topics) on the online platform. Each semester consisted of 20 lectures on different topics. The topics of the lectures did not vary between the terms.

During semesters one to three (from April 2013 to September 2014), the students were randomly assigned in a 1:2 ratio to the investigative group (with multiple-choice questions) or the control group (without multiple-choice questions). In the fourth semester (from October 2014 to March 2015), a switch was made to a 2:1 randomization for the investigative and control groups (Table 1). This change in randomization was necessary due to the growing distribution of the MC-questions. Completing the questions was not mandatory for the investigative group and did not affect the results of the final examination. The correct answers were shown to the students after each session had been completed, to provide feedback. The results and number of attempts to pass were not recorded. At the end of each semester, all of the students had to take part in a written examination containing 40 multiple-choice questions. A grade was assigned according to the number of points reached, with grade 1 representing the best result and grade 5 the poorest.

**Table 1:** Comparison between the two study cohorts

	Total		Without MC questions		With MC questions		*p* value	95% CI
	Mean	SD	Mean	SD	Mean	SD		
Semester	7.91	1.606	8.11	1.719	7.67	1.417	0.0001	– 0.678 to – 0.204
Attempt	1.04	0.211	1.04	0.227	1.03	0.19	0.438	– 0.044 to 0.19
Age [years]	24.68	2.804	24.71	2.928	24.63	2.557	0.859	– 0.963 to 0.804
Gender	M = 270		M = 148		M = 122		0.752	
	F = 405		F = 228		F = 177			

*CI* confidence interval, *MC* multiple-choice, *SD* standard deviation, *M* male, *F* female

**Fig. 1:**
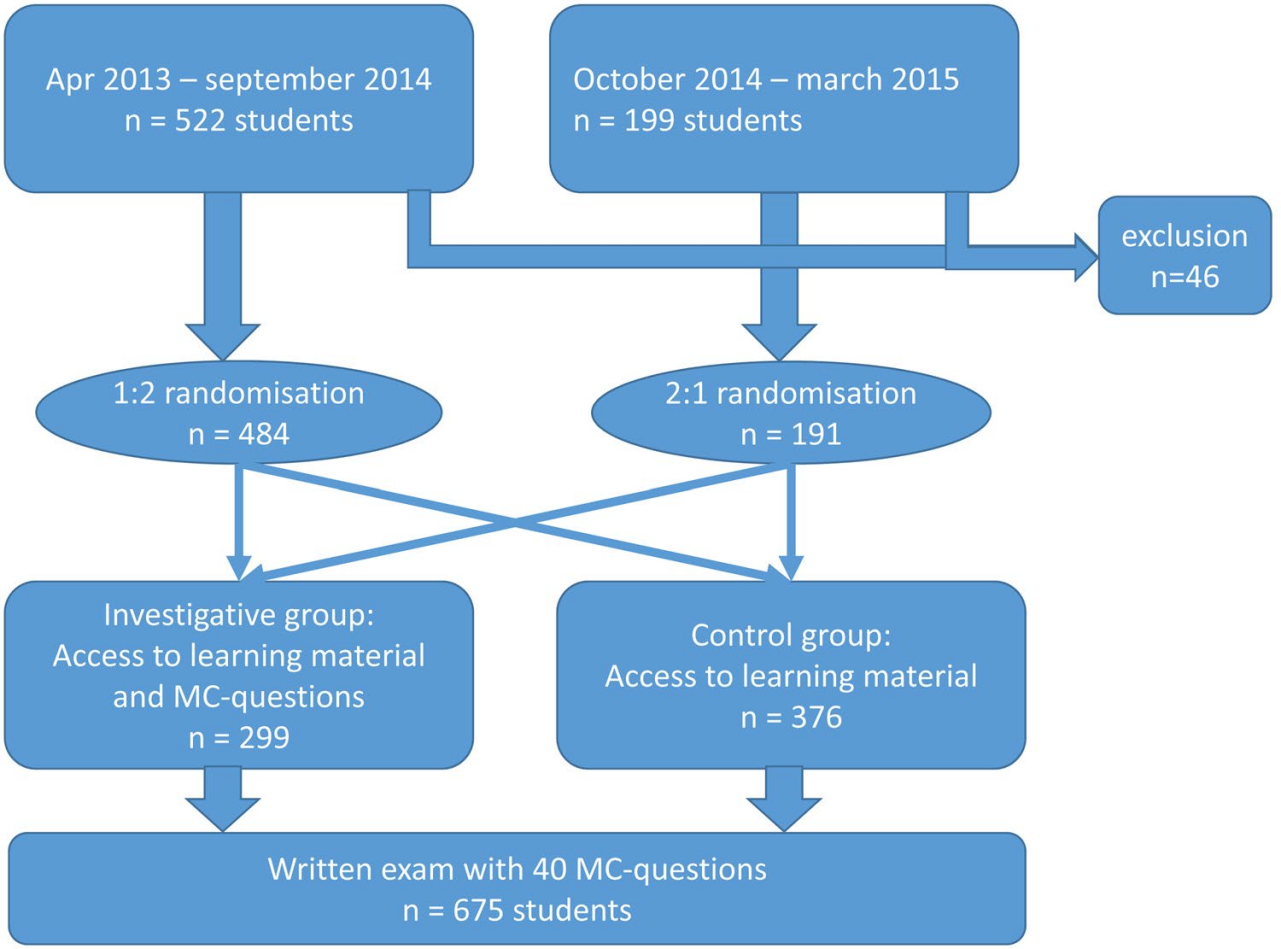
Flow chart of the study design

The aim of the study was to evaluate the impact of additional multiple-choice questions on the students’ results in the final examination at the end of the term. Figure 1 shows a flow chart of the study design.

### Data collection

All data used in this study were collected from the students attending the lecture-course on gynecology and obstetrics between April 2013 and March 2015 (i.e., four semesters) at Erlangen University Hospital, Friedrich Alexander University of Erlangen–Nuremberg. All students completed an online questionnaire with information about age, sex, semester, and number of attempts to pass.

### Statistical analysis

Data were acquired using Microsoft Excel 2016 (Microsoft Corporation, Redmond, Washington, USA), and statistical analyses were performed using SPSS Statistics for Windows, version 24 (IBM Corporation, Armonk, New York, USA). Fisher’s exact test was used to compare the characteristics of age, gender, number of attempts at the examination, and students’ study semester. One-way analysis of variance (ANOVA) was used to compare means for Likert-scaled items and point values. A *p* value of less than 0.05 was considered statistically significant.

## Results

### Descriptive statistics

A total of 721 students agreed to take part in the study. Of these, 46 were excluded from the final analysis—38 did not take part in the final written examination and eight were ERASMUS students (EuRopean Community Action Scheme for the Mobility of University Students, the student exchange program supported by the European Union).

This resulted in a total of 675 evaluable datasets for the final analysis (Table 1). The students’ mean age was 24.6 years (SD 2.804). Most of the students were in their seventh or eighth semester (mean 7.91, SD 1.606). The vast majority of students were attending the lecture-course in gynecology and obstetrics for the first time (mean attempts 1.04, SD 0.211). Only 21 students had to repeat the course and the written examination due to a failed previous attempt. Among the participants in the study, 60% (*n* = 405) were women and 40% (*n* = 270) men. In all, 299 (44.3%) participants had the opportunity to answer additional multiple-choice questions, while 376 (55.7%) were in the group without multiple-choice questions.

### Cohort comparison

Table 1 presents a comparison between the cohorts with and without multiple-choice questions. Students in the cohort with multiple-choice questions were in an earlier semester (7.67 vs. 8.11) than the students in the cohort without multiple-choice questions. No major differences were observed between the two groups in relation to age, gender, or number of attempts at the examination.

### Test results by cohort

Analysis of the results in the end-of-semester examination showed statistically significant differences between the two cohorts (Table 2). Students in the investigative group (with multiple-choice questions) scored significantly better grades (2.11 vs 2.49) and higher total scores (33 vs 31.31) than students in the control group (without multiple-choice questions) (*p* < 0.05).

**Table 2:** Comparison of examination results between the two study cohorts.

	Total		Without MC questions		With MC questions		*p* value	95% CI
	Mean	SD	Mean	SD	Mean	SD		
Grade	2.32	1.099	2.49	1.152	2.11	0.989	0.0001	– 0.546 to – 0.22
Points	32.06	4.947	31.31	5.3	33	4.291	0.0001	0.967 to 2.417

Presented are the grade and achieved points for the complete cohort and each group

*CI* confidence interval, *MC* multiple-choice, *SD* standard deviation

### Test results by cohort and semester

In a subsequent analysis, the test results for both study cohorts over the period of four semesters were examined (Table 3). For the 2013 summer term and 2014/2015 winter term, a statistically significant improvement in the examination results (overall points and grades) was observed in the investigative group (with multiple-choice questions) in comparison with the standard group (without multiple-choice questions). In the 2013/2014 winter term, significantly better results were observed for the overall points obtained in the investigative group (31.68 vs 30.18; *p* = 0.025). However, this did not lead to a statistically significant improvement in grading in the 2013/2014 winter term (2.52 vs 2.81; *p* = 0.073). No statistically significant differences between the two groups were observed in the third semester (2014 summer term).

**Table 3:** Comparison of cohorts by semester. Presented are the grade and achieved points for the complete cohort and each group, separately listed for each semester

2013 summer term								
	Total (*n* = 158)		With MC questions (*n* = 52)		Without MC questions (*n* = 106)		*p* value	95% CI
	Mean	SD	Mean	SD	Mean	SD		
Grade	2.36	1.322	1.98	1.18	2.55	1.353	0.008	– 0.982 to – 0.151
Points	31.35	6.91	32.96	6.502	30.56	6.995	0.035	0.167 to 4.643

2013/2014 winter term								
	Total (*n* = 164)		With MC questions (*n* = 56)		Without MC questions (*n* = 108)		*p* value	95% CI
	Mean	SD	Mean	SD	Mean	SD		
Grade	2.71	1.021	2.52	0.914	2.81	1.063	0.073	– 0.603 to 0.028
Points	30.69	4.42	31.68	3.629	30.18	4.714	0.025	0.19 to 2.815

2014 summer term								
	Total (*n* = 162)		With MC questions (*n* = 59)		Without MC questions (*n* = 103)		*p* value	95% CI
	Mean	SD	Mean	SD	Mean	SD		
Grade	2.19	1.031	2.22	1.068	2.17	1.014	0.791	– 0.293 to 0.385
Points	32.82	4.189	32.78	4.445	32.84	4.058	0.927	– 1.459 to 7.329

2014/2015 winter term								
	Total (*n* = 191)		With MC questions (*n* = 132)		Without MC questions (*n* = 59)		*p* value	95% CI
	Mean	SD	Mean	SD	Mean	SD		
Grade	2.08	0.917	1.94	0.845	2.39	1	0.003	– 0.747 to – 0.154
Points	33.18	3.454	33.68	3.149	32.07	3.855	0.006	0.479 to 2.749

Significance was not present in the summer term 2014

*CI* confidence interval, *MC* multiple-choice, *SD* standard deviation

### Differences in test results by cohort during the course of the study

**Fig. 2:**
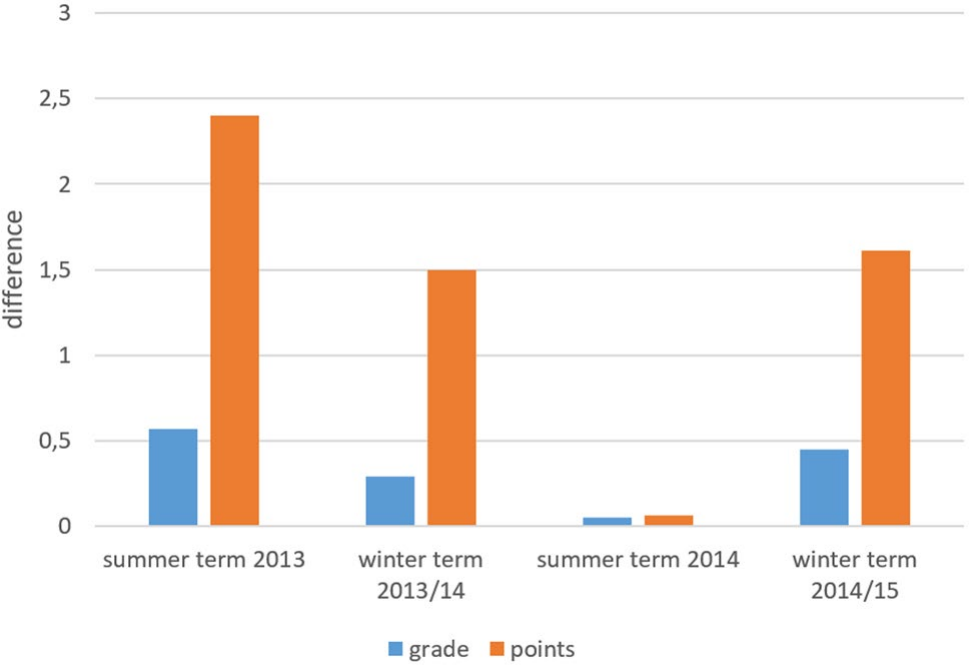
Differences between the groups (grade and points achieved) during the study

Another analysis compared the differences in test results in the two cohorts over the course of the study (Fig. 2). Starting with the 2013 summer term, the differences between the two groups diminished until the 2014 summer term, in which no statistically significant differences were noted. However, a difference between the two study cohorts was again found in the 2014/2015 winter term.

## Discussion

This report describes the first randomized study on the implementation of voluntary multiple-choice questions in a classic university lecture-course. Students who had had an opportunity to answer voluntary multiple-choice questions on each topic performed better statistically in a written examination based on multiple-choice questions at the end of the semester. The findings thus have important implications for lecturers and students.

The findings of the study are plausible, as the phenomenon of test-enhanced learning is well-known and established. Multiple-choice questions provide a basis for recalling curricular material and increase knowledge retention. The findings are consistent with previous research on test-enhanced learning and other methods of intensifying the retention of knowledge [10]. This study adds to the known and existing research on ways of improving lectures. Studies have also shown that undergraduate education through lectures can be improved using audience response, presenter–learner interaction, and clinically relevant material [14–18].

Research suggests that test-enhanced learning is most effective when active production of knowledge is used (questions that require the student to frame an answer are associated with better results than multiple-choice questions alone), when tests are separated over time, and when feedback is given after a certain time after the test [5]. Other studies have not observed any impact on medical students’ performance, although an effect of additional testing on motivation was noted [6].

The advantage of the present study is that it demonstrates the effect over time. The phenomenon that the positive effect decreased over time in the study was analyzed. It was found that the multiple-choice questions were answered together in learning groups, which led to even students in the group without multiple-choice questions having access to the additional questions. It was also noted that the questions were published on social media platforms such as closed Facebook groups and WhatsApp groups. Over time, therefore, the groups could not be clearly divided as originally randomized. After the randomization mode was changed to include more students in the investigation group, the need to distribute the questions obviously decreased, and only the most motivated students in the control group tried to obtain access to the multiple-choice questions. This explains the statistically significant difference in the last semester analyzed.

The study has several weaknesses. First, it was not recorded whether the multiple-choice questions were answered during the semester, or at what time point. The number of attempts and the actual results of the additional test were also not recorded so that the effect of these aspects could not be evaluated. Second, only one institution supplied the multiple-choice questions, and the results can therefore not be generalized. And third, each lecture-course was given by a different lecturer. The lectures were the same in each semester, but comparability between the different lecturers is not possible.

Finally, yet importantly, the comments provided in the students’ evaluation of the lecture-courses at the end of each semester were overwhelming. More than 40 positive comments on the opportunity to answer extra questions were received every semester.

## Conclusions

To the best of our knowledge, this is the first randomized study examining the implementation of voluntary multiple-choice questions in a lecture-course for undergraduate medical students in gynecology and obstetrics.

Providing multiple-choice questions to accompany lectures is an easy tool for improving students’ results in the final examination. On the basis of the data obtained, multiple-choice questions are now routinely offered for every lesson to improve the students’ test results. The implementation of multiple-choice questions is feasible and easily established for everyone. No change in the structure of the lecture-courses is needed. As a side effect, the students’ routine evaluation of the lectures improved.

The effect over time should be considered for evaluation. For example, the effect can be measured during practical training 1 year later [19]. It may be expected that the effect of test-enhanced learning will not last for as long as has been reported in other studies [13].

As a future prospect, a combination of a pause procedure with online multiple-choice questions is an interesting field, since these two easy-to-implement tools that lead to known improvement in students’ performance may have an even greater effect. The impact of these methods on long-term knowledge retention should also be evaluated.
