# Correction to: Introducing multiple-choice questions to promote learning for medical students: effect on exam performance in obstetrics and gynecology

**DOI:** 10.1007/s00404-021-06112-9

**Published:** 2021-06-21

**Authors:** Sebastian M. Jud, Susanne Cupisti, Wolfgang Frobenius, Andrea Winkler, Franziska Schultheis, Sophia Antoniadis, Matthias W. Beckmann, Felix Heindl

**Affiliations:** Department of Gynecology and Obstetrics, Erlangen University Hospital, Friedrich Alexander University of Erlangen–Nuremberg, Universitätsstrasse 21–23, 91054 Erlangen, Germany

## Correction to: Archives of Gynecology and Obstetrics (2020) 302:1401–1406 10.1007/s00404-020-05758-1

The article “Introducing multiple-choice questions to promote learning for medical students: effect on exam performance in obstetrics and gynecology” written by Sebastian M. Jud, Susanne Cupisti, Wolfgang Frobenius, Andrea Winkler, Franziska Schultheis, Sophia Antoniadis, Matthias W. Beckmann and Felix Heindl was originally published electronically on the publisher’s internet portal on August 31, 2020 without open access. With the author(s)’ decision to opt for Open Choice the copyright of the article changed to © The Author(s) 2020 and the article is forthwith distributed under a Creative Commons Attribution 4.0 International License, which permits use, sharing, adaptation, distribution and reproduction in any medium or format, as long as you give appropriate credit to the original author(s) and the source, provide a link to the Creative Commons licence, and indicate if changes were made. The images or other third-party material in this article are included in the article’s Creative Commons licence, unless indicated otherwise in a credit line to the material. If material is not included in the article’s Creative Commons licence and your intended use is not permitted by statutory regulation or exceeds the permitted use, you will need to obtain permission directly from the copyright holder. To view a copy of this licence, visit http://creativecommons.org/licenses/by/4.0/. Open Access funding enabled and organized by Projekt DEAL.

**Open Access**: The article is forthwith distributed under a Creative Commons Attribution 4.0 International License, which permits use, sharing, adaptation, distribution and reproduction in any medium or format, as long as you give appropriate credit to the original author(s) and the source, provide a link to the Creative Commons licence, and indicate if changes were made. The images or other third-party material in this article are included in the article’s Creative Commons licence, unless indicated otherwise in a credit line to the material. If material is not included in the article’s Creative Commons licence and your intended use is not permitted by statutory regulation or exceeds the permitted use, you will need to obtain permission directly from the copyright holder. To view a copy of this licence, visit http://creativecommons.org/licenses/by/4.0/.

